# Interactions Between SNP Alleles at Multiple Loci and Variation in Skin Pigmentation in 122 Caucasians

**Published:** 2007-09-06

**Authors:** Sumiko Anno, Takashi Abe, Koichi Sairyo, Susumu Kudo, Takushi Yamamoto, Koretsugu Ogata, Vijay K. Goel

**Affiliations:** 1 School of Engineering, Shibaura Institute of Technology, Koto-ku, Tokyo, Japan.; 2 The laboratory for Research and Development of Biological Databases, National Institute of Genetics, Mishima-shi, Shizuoka, Japan.; 3 Faculty of Medicine, The University of Tokushima, Tokushima-shi, Tokushima, Japan.; 4 Shimadzu Biotech, Shimadzu Corporation, Kyoto-shi, Kyoto, Japan.; 5 Department of Bioengineering, College of Engineering, University of Toledo, Toledo, Ohio, U.S.A.

**Keywords:** Single nucleotide polymorphisms (SNPs), Polygene, Environmental adaptability

## Abstract

This study was undertaken to clarify the molecular basis for human skin color variation and the environmental adaptability to ultraviolet irradiation, with the ultimate goal of predicting the impact of changes in future environments on human health risk. One hundred twenty-two Caucasians living in Toledo, Ohio participated. Back and cheek skin were assayed for melanin as a quantitative trait marker. Buccal cell samples were collected and used for DNA extraction. DNA was used for SNP genotyping using the Masscode™ system, which entails two-step PCR amplification and a platform chemistry which allows cleavable mass spectrometry tags. The results show gene-gene interaction between SNP alleles at multiple loci (not necessarily on the same chromosome) contributes to inter-individual skin color variation while suggesting a high probability of linkage disequilibrium. Confirmation of these findings requires further study with other ethic groups to analyze the associations between SNP alleles at multiple loci and human skin color variation. Our overarching goal is to use remote sensing data to clarify the interaction between atmospheric environments and SNP allelic frequency and investigate human adaptability to ultraviolet irradiation. Such information should greatly assist in the prediction of the health effects of future environmental changes such as ozone depletion and increased ultraviolet exposure. If such health effects are to some extent predictable, it might be possible to prepare for such changes in advance and thus reduce the extent of their impact.

## Introduction

### Background

The increasing pace of human industrial technology has powerfully contributed to environmental change. For example, industrial activity has sped up the rate of ozone depletion and led to an increased exposure to ultraviolet (UV) rays—conditions to which humans have not had to adapt. Thus, human survival could ultimately depend on an understanding of the adaptation that will be required.

There are two important components to understanding the adaptation that will be required. First, it is important to understand the dynamics of the human genome, which encodes the complex human phenotype. Second, it is important to understand how the environment exerts pressures and effects on the genome such that it helps determine human traits. Genetic and environmental factors are both part of an elaborate feedback mechanism whereby the human adaptive form reacts to environmental stimuli with internal adjustments (Anno et al. 2006).

### Human skin color as an environmental adaptation

Humans have adapted to complicated and challenging environments by evolving new traits and abilities. Human skin color variations, accomplished by increases and decreases in melanin, represent an environmental adaptation to different levels of exposure to UV rays (John et al. 2003; Aoki, 2002). For example, people indigenous to Northern Europe have pale skin, while people indigenous to Africa have dark skin. At low latitudes, melanin production is increased to protect against repeated UV irradiation. At high latitudes, melanin production is decreased, which increases the body’s ability to synthesize vitamin D and offers a variety of health benefits, including protection against rickets (osteomalacia).

### Mechanism of melanin formation

Human cutaneous pigmentation is primarily determined by melanin (the black/brown eumelanin and the yellow/red pheomelanin), which is synthesized in the melanosome (an organelle contained within melanocytes). The mechanism of melanin formation is as follows. UV irradiation of human melanocytes triggers the binding of α-MSH to the G-protein-coupled receptor melanocortin 1 receptor (MC1R), activating adenylate cyclase and increasing cAMP formation. The increase in intracellular cAMP ultimately stimulates the expression of enzymes important in eumelanin biosynthesis, including tyrosinase, which catalyzes the oxidation of tyrosine to dopaquinone. Dopaquinone is the last common precursor of eumelanin and pheomelanin, whose fate is largely determined by the signaling state of the MC1R. The binding of agouti to MC1R activates the signal transduction events that lead to pheomelanin synthesis (Akey et al. 2001; Rees, 2003).

Human skin color is determined by the size, shape, distribution, and chemical composition of eumelanin and pheomelanin, not the number of melanocytes. Differences in human pigmentation are understood to be largely the result of differences in eumelanin content, which is determined by melanin productivity and is genetically controlled (Akey et al. 2001; Rees, 2003).

### Skin color diversity due to DNA polymorphism

Although approximately 99% of human DNA sequences are reported to be identical across the human population, variations in DNA sequence still exert an important impact on human diversity, affecting disease risk, drug responsiveness (including adverse drug responses), and human phenotypes (Akey et al. 2001; Rees, 2003). A single nucleotide polymorphism (SNP) is a genetic variation occurring ≥1% of the population. The ability to establish the functional relationships of genetic variations offers the possibility of individualized treatment, disease prevention, and an elucidation of the evolutionary and biophysical functions that characterize human phenotypes.

Several studies of skin pigmentation have reported polymorphisms at loci such as MC1R, oculocutaneous albinism II (P), and agouti signaling protein (Agouti) to be associated with skin color variation. Still, human skin color variation is thought to likely be controlled by interactions between SNP alleles at multiple loci (Flanagan et al. 2000; Akey et al. 2001; Peng et al. 2001; Voisey et al. 2001; Nakayama et al. 2002; John et al. 2003; Rees, 2003; Naysmith et al. 2004).

### Objectives

The ozone layer prevents the most harmful UVB wavelengths from passing through the Earth's atmosphere. The depletion of ozone by chlorofluorocarbons (CFCs) has therefore become an important environmental issue. Increased UV exposure is suspected to have a variety of biological consequences, including an increased incidence of skin cancer, damage to plants, and reduction of plankton populations in the ocean photic zone. Although variations in human skin color occur are known to occur in connection with environmental factors such as UV radiation, the molecular basis for the genetic background of human skin color is still unclear.

This study was undertaken to clarify not only the molecular basis for the genetic background of human skin color variation but also human adaptability from the perspective of human evolution. To address these issues and to predict how environmental changes such as increased UV rays due to ozone depletion might influence human health in the future, and to consider the room for adaptation by human beings to the changing ecosystem, we investigated how SNP alleles at multiple loci contribute to variation in skin pigmentation. Using SNP genotyping via the Masscode™ system we determined the molecular basis for the skin color genetic background of 122 Caucasians.

## Materials and Methods

### Sample collection and melanin measurement

One hundred twenty-two Caucasians living in Toledo, Ohio participated. Sample collection and melanin measurement were conducted in accordance with the protocol approved by the Human Subjects Research Committee of the University of Toledo (Research project # 205–008, Approved by Human Subjects Research Committee of University of Toledo).

Subjects gave informed consent for the collection of buccal samples. Samples were anonymously coded. The melanin skin pigmentation index was determined using the Mobile Mexameter MSC100/ MX18 (Integral Corporation, Shinjuku-ku, Tokyo, Japan), which uses photodiode arrays to measure the intensity of particular light wavelengths. Two measurements were obtained from the back, as an index of inherent skin color influenced by genetic factors, and two measurements were obtained from the cheek, as an index of modified skin color influenced by environmental factors.

### DNA extraction and genomic DNA amplification

DNA was extracted from the samples using the DNA extraction kit ISOHAIR (NIPPON GENE Co., Ltd., Chiyoda-ku, Tokyo, Japan). In order to provide sufficient genomic DNA for SNP genotyping, whole genomic DNA was amplified using the REPLI-g Kit (QIAGEN K. K., Chuo-ku, Tokyo, Japan).

### SNP genotyping with the Masscode^™^ system

#### 1) Primer design and polymerase chain reaction

High-throughput SNP genotyping with the Mass-code^TM^ system was employed (Kokoris et al. 2000; Ogata et al. 2002). SNP genotyping was performed for seven genes involved in melanin synthesis and pigmentation. Two SNP-spanning external primers and two hemi-nested allele-specific primers were designed for the identification of molecular alternations at 20 SNPs (rs819136, rs1129414, rs2075508, rs10960756, rs3793976, rs2298458, rs3212363, rs1805008, rs3212371, rs2279727, rs4778182, rs1800419, rs2311843, rs1800414, rs1800404, rs7623610, rs704246, rs16964944, rs1724577, and rs4776053) in the ASIP, TYRP1, TYR, MC1R, OCA2, MITF, and MYO5A genes.

The Masscode SNP discrimination assay is a solution-based technique that uses a two-step polymerase chain reaction (PCR) process to selectively amplify and CMST-label the SNP alleles present in the template DNA. The primary external PCR is a standard amplification that uses a primer pair spanning the SNP of interest. The process begins with the addition of the master mix and primer to a 96-well PCR plate containing the dried DNA templates (5 ng genomic DNA). Our study used a touchdown PCR thermocycling method consisting of an initia1 90-s 92 °C denaturation, followed by 15 cycles of denaturing (15-s at 92 °C), annealing (15-s at 65 °C), and extension (60-s at 72 °C), with a l °C reduction of the annealing temperature for each successive cycle. An additiona1 30 cycles of denaturating (15-s at 92 °C), annealing (15-s at 50 °C), and extension (60-s at 72 °C), fo1lowed by a single 5-min extension (72 °C), completed the process. After the first PCR, the PCR products were subjected to 2% agarose gel electrophoresis to verify that the expected single-band product was generated. The verified PCR products were purified with ExoSAP-IT (Amersham). The purified PCR products, two hemi-nested allele-specific primers, and two universally tagged Masscode oligonucleotides were used for the allele discrimination assay. Each tag was covalently attached to the 5′ end of an oligonucleotide via a photolabile linker. Tagged oligonucleotides were used as a primer in an allele-specific PCR SNP discrimination assay, in which multiple SNP alleles at a particular locus were amplified by PCR using specific primers with tags differing in molecular weight (Kokoris et al. 2000; Ogata et al. 2002).

#### 2) Purification and Masscode^TM^ Tag detection

Following PCR, the SNP-specific PCR products were passed through a QIAquick 96 silica-based filter membrane to remove unincorporated tagged primers. The tags were cleaved from the purified PCR products by exposure to a 254-nm mercury lamp and analyzed using an optimized Agilent 1100 single quadrupole mass spectrometer, which detects the tagged species present in the sample. The presence of a particular tag indicates the presence of the corresponding SNP allele in the genomic DNA used as the PCR template. Genotype data are reported in a comma-delimited flat-file format that contains the SNP and sample identifiers for each allele call. Alleles are reported using a binary nomenclature in which l represents wild-type alleles and 2 represents variant alleles. The SNP allele was classified into three types: wild-type homozygous, variant-type homozygous, and heterozygous. Thus, a homozygous wild-type allele is designated 1,1, and a heterozygote allele is designated 1, 2 (Kokoris et al. 2000; Ogata et al. 2002).

### Statistical analysis

The genotype and allele frequencies for 20 SNPs in 122 Caucasians were calculated with the data obtained from the SNP genotyping.

For each back sample we calculated the *mean value of melanin* ± *2σ* and used the result to determine two groups (low melanin: n = 59, melanin ≤94.8; high melanin: n = 39; mean ≥137.2) based on constitutive skin color. To examine the contribution of non-random associations of SNP alleles at multiple loci (not necessarily on the same chromosome) to skin color variation (i.e. low/high melanin content), we examined the associations of 20 SNP alleles at various loci in the genome (in candidate genes) by use of linkage disequilibrium (LD), which can serve as a measure of gene-gene interaction between unlinked loci (Zhao et al. 2006). The p value of LD was analyzed by application of a χ^2^ test; statistical significance was set at 0.05. Combinations of SNP alleles at multiple loci under LD were jointly tested for association with low/high melanin by performing a χ^2^ test for independence. Only data under the condition of Hardy-Weinberg equilibrium were used in the analysis.

## Results

**Figure 1.**
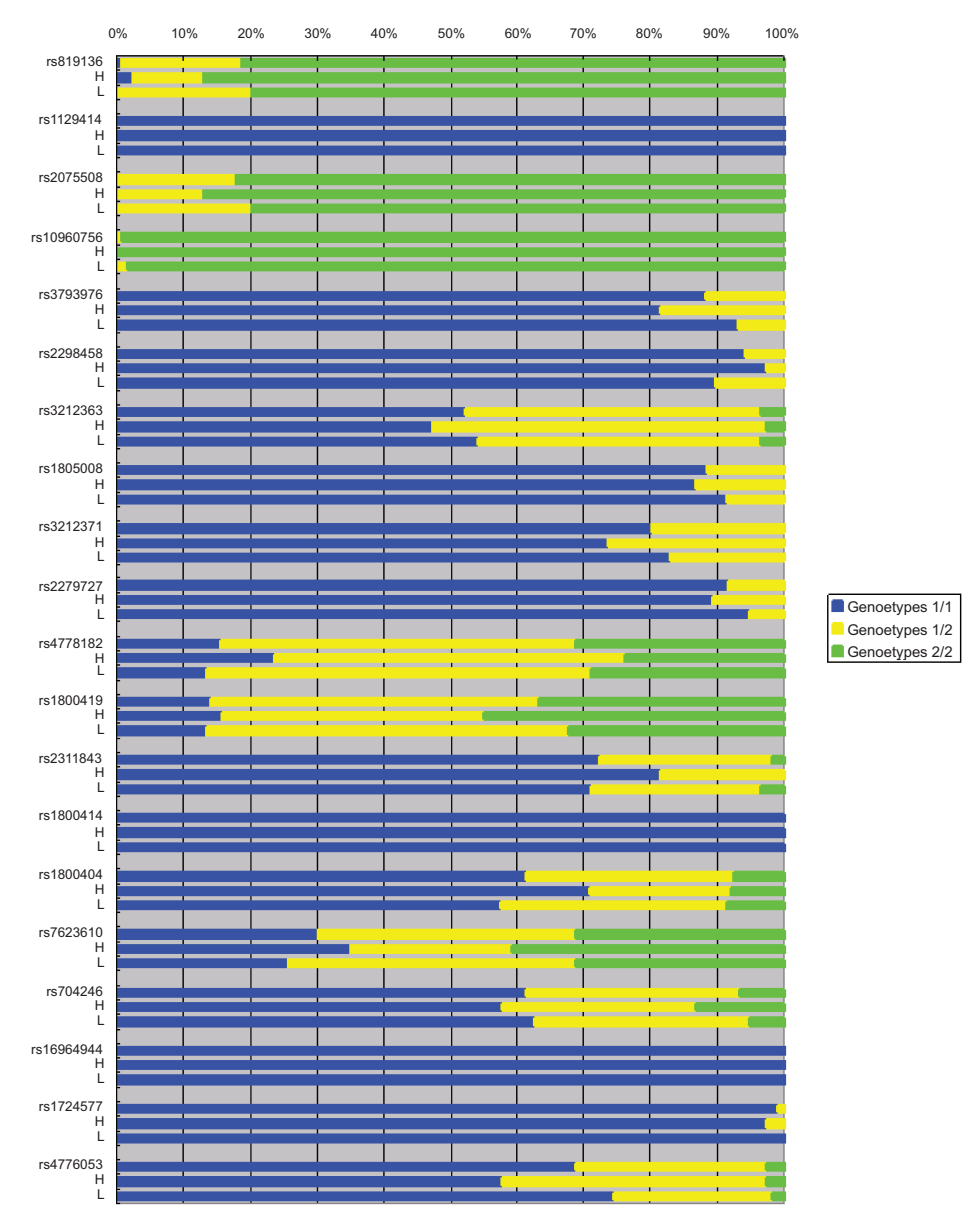
Genotype frequencies for 20 SNPs from samples with high and low melanin values. #rs: all subjects; H: high melanin value; L: low melanin value.

**Figure 2.**
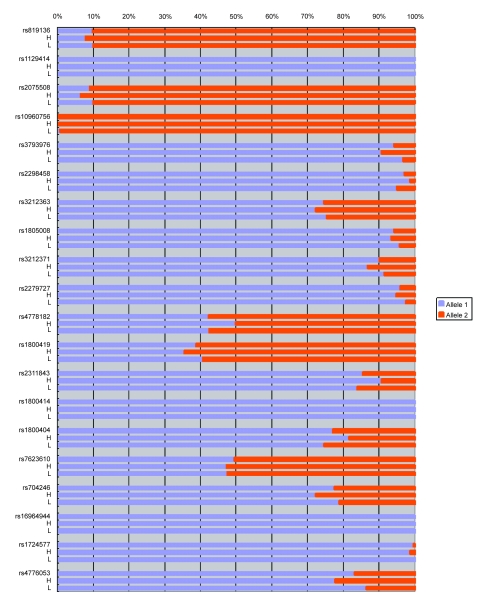
Allele frequencies for 20 SNPs from samples with high and low melanin values. #rs: all subjects; H: high melanin value; L: low melanin value.

Table 1, Figure 1, and Figure 2 show the genotype and allele frequencies for the 20 SNPs. The LD coefficients (D) for the 20 SNPs are shown in Table 2. The p values obtained from the χ^2^ test are shown in Table 3. A p value <0.05 indicates LD.

Combinations of the SNP alleles at multiple loci under LD were constructed for the low and high melanin groups. Low melanin was associated with the allele combination rs819136G/rs3212371A/ rs2279727A/rs2311843T (p = 0.0016). This combination of alleles might be indicative of an inhibitory factor responsible for low melanin synthesis. The MC1R is activated by α-MSH and antagonized by agouti signaling protein (ASIP). When MC1R is inactive, dopaquinone is preferentially converted into pheomelanin, which is responsible for light skin color and low level melanin. Thus, rs819136G, which is in the gene for ASIP, can lead to this result. There was a significant association between high melanin and the allele combination rs3793976T/rs2298458G/rs3212363T/ rs1805008C (p = 0.0269). This combination of alleles appears to promote eumelanin synthesis, resulting in a dark skin color. The ASIP, TYRP1, TYR, MC1R, OCA2, MITF, and MYO5A genes have been identified that are likely to play a role in normal pigment variation, either by controlling the rate and type of melanin produced, or by exerting an effect on the melanin biosynthetic pathway (John et al. 2003). The rs3793976T and rs2298458G alleles in the TYR gene produce enzymes which act in the melanin biosynthetic pathway and contribute to this result.

## Discussion

Human skin color variation is assumed to result from the interaction of a number of genes, with each gene having a small impact on the final effect. A gene-gene interaction between SNP alleles at multiple loci is an important but also complex model concept. Identifying both the relevant genes and the variable loci within them that determine inter-individual skin color variation is one of the most difficult challenges of this type. Most approaches of related studies have chosen to evaluate single genes (the haplotype approach). This approach ignores the possibility that the effects of genetic units consisting of multi loci influence or even control the variations. Furthermore, classical genetic analyses limit the power to detect interaction, essentially ruling out such interaction before examining whether it is appropriate to make this determination. Such analyses could readily lack a basis for a biological interpretation of gene-gene interaction (Zhao et al. 2006). Therefore, we conducted an investigation of a gene-gene pattern of interaction between SNP alleles at multiple loci (not necessarily on the same chromosome), which contributes to the inquiry into inter-individual skin color variation by use of LD, which can serve as a measure of gene-gene interaction between unlinked loci (Zhao et al. 2006).

Our results show that gene-gene interaction between SNP alleles at multiple loci contributes to inter-individual skin color as well as melanin variation—indicating a high possibility of LD structure—despite the fact that the alleles are not on the same chromosome. These results were obtained from 122 Caucasians. Undoubtedly, a greater number of subjects will be needed to obtain more robustly accurate results. Still, since a SNP is a genetic variation occurring in ≥1% of the population, 122 subjects is a number sufficient to meet at least the preliminary conditions. Similar investigations will also be needed with samples from non-Caucasians in order to determine whether gene-gene interaction between SNP alleles at multiple loci contributes to inter-individual skin color variations or racial differences. These results should be understood to be preliminary. There is a need to develop analytical methods for rigorously testing gene-gene interactions and identifying the combinations of variable loci that determine the phenotypic variations. The ultimate overarching goal of this work is to eventually use remote sensing data to clarify the relationship of the atmospheric environment and SNP allelic frequency, and to investigate environmental impacts on the human adaptability to UV irradiation. The resulting data should prove to be of great help in predicting the impact of environmental changes on health risk, such as high UV exposure secondary to ozone depletion.

## Figures and Tables

**Table 1.** Genotype and allele frequencies for 20 SNPs.

**#rs**	**rs819136**			**rs1129414**			**rs2075508**			**rs10960756**			**rs3793976**		

**Allele Type**	**A/G**			**A/C**			**C/T**			**A/G**			**G/T**		

**Melanin Value**	**all**	**high**	**low**	**all**	**high**	**low**	**all**	**high**	**low**	**all**	**high**	**low**	**all**	**high**	**low**
Genotypes 1/1	0.01	0.03	0.00	1.00	1.00	1.00	0.00	0.00	0.00	0.00	0.00	0.00	0.88	0.82	0.93
Genotypes 1/2	0.18	0.11	0.20	0.00	0.00	0.00	0.18	0.13	0.20	0.01	0.00	0.02	0.12	0.18	0.07
Genotypes 2/2	0.81	0.87	0.80	0.00	0.00	0.00	0.82	0.87	0.80	0.99	1.00	0.98	0.00	0.00	0.00
Alleles 1	0.10	0.08	0.10	1.00	1.00	1.00	0.09	0.07	0.10	0.00	0.00	0.01	0.94	0.91	0.97
Alleles 2	0.90	0.92	0.90	0.00	0.00	0.00	0.91	0.93	0.90	1.00	1.00	0.99	0.06	0.09	0.03

**#rs**	**rs2298458**			**rs3212363**			**rs1805008**			**rs3212371**			**rs2279727**		

**Allele Type**	**G/T**			**A/T**			**C/T**			**A/G**			**A/C**		

**Melanin Value**	**all**	**high**	**low**	**all**	**high**	**low**	**all**	**high**	**low**	**all**	**high**	**low**	**all**	**high**	**low**

Genotypes 1/1	0.94	0.97	0.90	0.52	0.47	0.54	0.89	0.87	0.92	0.80	0.74	0.83	0.92	0.89	0.95
Genotypes 1/2	0.06	0.03	0.10	0.44	0.50	0.42	0.11	0.13	0.08	0.20	0.26	0.17	0.08	0.11	0.05
Genotypes 2/2	0.00	0.00	0.00	0.03	0.03	0.03	0.00	0.00	0.00	0.00	0.00	0.00	0.00	0.00	0.00
Alleles 1	0.97	0.99	0.95	0.75	0.72	0.75	0.94	0.93	0.96	0.90	0.87	0.92	0.96	0.95	0.97
Alleles 2	0.03	0.01	0.05	0.25	0.28	0.25	0.06	0.07	0.04	0.10	0.13	0.08	0.04	0.05	0.03

**#rs**	**rs4778182**			**rs1800419**			**rs2311843**			**rs1800414**			**rs1800404**		

**Allele Type**	**A/G**			**C/T**			**C/T**			**A/G**			**A/G**		

**Melanin Value**	**all**	**high**	**low**	**all**	**high**	**low**	**all**	**high**	**low**	**all**	**high**	**low**	**all**	**high**	**low**

Genotypes 1/1	0.16	0.24	0.14	0.14	0.16	0.14	0.73	0.82	0.71	1.00	1.00	1.00	0.61	0.71	0.58
Genotypes 1/2	0.53	0.53	0.58	0.49	0.39	0.54	0.26	0.18	0.25	0.00	0.00	0.00	0.31	0.21	0.34
Genotypes 2/2	0.31	0.24	0.29	0.37	0.45	0.32	0.02	0.00	0.03	0.00	0.00	0.00	0.07	0.08	0.08
Alleles 1	0.42	0.50	0.42	0.39	0.36	0.41	0.85	0.91	0.84	1.00	1.00	1.00	0.77	0.82	0.75
Alleles 2	0.58	0.50	0.58	0.61	0.64	0.59	0.15	0.09	0.16	0.00	0.00	0.00	0.23	0.18	0.25

**#rs**	**rs7623610**			**rs704246**			**rs16964944**			**rs1724577**			**rs4776053**		

**Allele Type**	**A/G**			**C/T**			**A/G**			**G/T**			**C/T**		

**Melanin Value**	**all**	**high**	**low**	**all**	**high**	**low**	**all**	**high**	**low**	**all**	**high**	**low**	**all**	**high**	**low**

Genotypes 1/1	0.30	0.35	0.26	0.61	0.58	0.63	1.00	1.00	1.00	0.99	0.97	1.00	0.69	0.58	0.75
Genotypes 1/2	0.39	0.24	0.43	0.32	0.29	0.32	0.00	0.00	0.00	0.01	0.03	0.00	0.29	0.39	0.24
Genotypes 2/2	0.31	0.41	0.31	0.07	0.13	0.05	0.00	0.00	0.00	0.00	0.00	0.00	0.02	0.03	0.02
Alleles 1	0.50	0.47	0.47	0.77	0.72	0.79	1.00	1.00	1.00	1.00	0.99	1.00	0.83	0.78	0.86
Alleles 2	0.50	0.53	0.53	0.23	0.28	0.21	0.00	0.00	0.00	0.00	0.01	0.00	0.17	0.22	0.14

**Note:** 1) In case of allele type ○/□, 1/1→○/○, 1/2→○/□, 2/2→□/□.

2) Melanin Value: all→all subjects, high→the group with high value of melanin, low→the group with low value of melanin

**Table 2.**
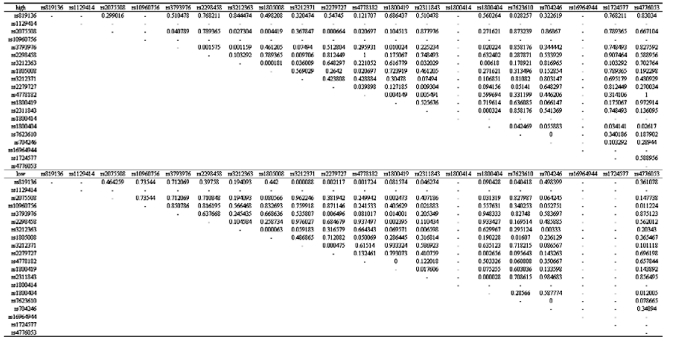
Linkage disequilibrium coefficients (D) between 20 SNPs.

**Table 3.**
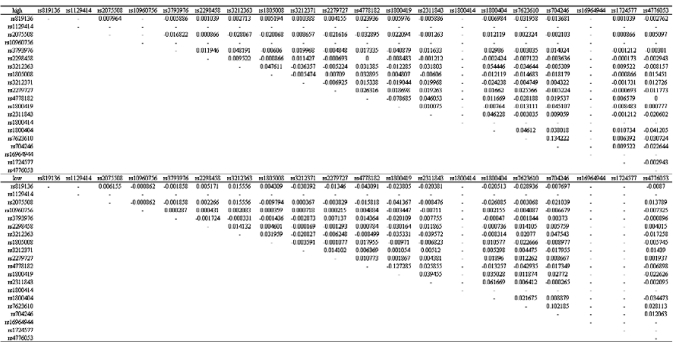
Results of the χ^2^ test of linkage disequilibrium.

**Note:** The p value of less than 0.05 was considered statistically significant and indicated that the SNPs were in linkage disequilibrium (LD).
